# Connecticut Retreat for the Insane

**Published:** 1841-03

**Authors:** 


### THE WESTERN JOURNAL.

Vol. III.—No. III.

LOUISVILLE, MARCH 1, 1841.

### CONNECTICUT RETRFAT FOR THE INSANE.

We have received from Dr. Brigham a copy of the Report of the Directors of this noble institution for 1840, and seldom have we contemplated the details of a paper with the same satisfaction. From a table exhibiting the number of admissions, recoveries, and deaths, annually, during a period of fifteen years, the proportion of cures, including old and confirmed, as well as recent cases, is shown to be more than fifty per cent. The total number of admissions being 1,001, the total number of recoveries was 563. But of the 537 recent cases admitted, 451 were cured. To the humanity, judgment and skill of the officers of the Retreat no words could testify with the force of these figures. They speak a language cheering to the heart of every physician and philanthropist. Yet, it is but sixteen years since, it is stated in the Report, that the proposition to establish an institution, which has proved itself thus capable of relieving human anguish in one of its most dreadful forms, was coldly received. By some the utility of the scheme was questioned, and by others its necessity was denied. For sixteen years it has been restoring to reason and to society more than half of all the insane committed to its charge; and of those in whom the disease has not become inveterate by time and bad management, more than four-fifths. The process by which results, so unlike those of lunatic asylums under the old *regime*, have been brought about, is described in the following paragraphs:

"It has been a cardinal principle with the Directors from the first establishment of the Retreat, to provide with parental interest for the wants of its unfortunate inmates. They have aimed to place it in charge of men dlstinguished for their skill, humanity and integrity, and require of them the unceasing exercise of these virtues.

"The physician is nominated by a committee of the Medical Society, and approved by the Directors. He is the head of the institution—is responsible for its management—conducts the medical and moral treatment of the patients, and maintains a regular correspondence with their friends.

"The importance of religious instruction as part of the system of moral treatment of the insane, has not been overlooked. Those of the patients who are capable of joining in religious services, assemble every evening, when the chaplain reads a portion of the sacred scriptures and conducts the devotions of the family. On Sunday he preaches to the assembled household, and often, during the week, presents the truths of christianity and its consolation to those who are care-worn, anxious, and dejected. Much good has resulted from this practice, and much credit is due to the gentleman, who has, with uncommon discretion and sound judgment, performed this sacred duty.

"A steward is appointed to make provision for the wants of the institution and of the patients, with the advice of the physician, and to perform whatever duties are appropriate to his station. It is his business also, under the direction of the superintendent and managers, to see that the buildings are kept in good repair—that the grounds are well cultivated, and that the quarterly dues for the board of the patients are collected. He is required, every month, to render to one of the Managers, a detailed statement of his receipts and expenditures.

"The matron of the institution has usually been the wife of the steward. She is required to devote much time to the female patients and their attendants—to see that the former are furnished with whatever is necessary for their comfort, and that the latter are faithful and kind in the performance of their duties. The matron has also been required to superintend the domestic concerns of the household, and to see that the rooms, the beds, the clothing, and the food of the pdtients are never neglected. These multiplied duties were too much for one person to perform, and it was consequently determined to appoint a house-keeper, to take charge of the domestic concerns of the institution, and require the matron to devote her attention exclusively to the welfare of the female patients.

"The early patrons of the Retreat were especially anxious to guard against every possible abuse. They directed that a committee of medical men should be chosen every year, authorized to visit dhe institution, to inspect its records, and inquire into its usuages, with instructions, if they discovered any thing wrong, to report it. It is the business of this committee to visit the Retreat every month—to assure themselves that is well conducted—that the patients are treated with uniform kindness, and that no reasonable exertions are spared to promote their recovery. A committee of ladies is annually appointed, whose attention is mainly directed to the condition of the female patients. Their visits are always welcome, and their suggestions have been the source of important practical improvements.

"It has always been our endeavor to make the Retreat an eligible place of residence—to allow the patients every liberty consistent with their safety, and to subject them to no severe restraint. But in order to secure this desirable object, it is necessary to be provided with a competent number of attendants, who, by assiduity and vigilance, shall supply the place of bolts and keys It is their business to walk or ride with the patients—to engage with them in their various schemes of recreation, and, if possible, to induce them to engage in some useful employment. The expense of supporting patients is materially increased by the plan to which we have alluded, but when it is remembered that it is attended with greater success—that it is more humane and more in accordance with the practice of the best asylums in the country, it will be admitted that no other, at least no better course could be adopted."

Dr. Brigham, well known for his professional industry and attainments, and especially prized at home for his umblemished moral character, has lately been elected superintendent of the institution. We feel assured, with the Directors, that his duties will be ably and faithfully performed—that his exertions will be crowed with success—and that he will be an instrument of great good to the insane, and of extending the fame and usefulness of the Retreat. Y.

